# Metal-free enantiomorphic perovskite [dabcoH_2_]^2+^[H_3_O]^+^Br^−^_3_ and its one-dimensional polar polymorph

**DOI:** 10.1107/S2052252522006406

**Published:** 2022-07-16

**Authors:** Armand Budzianowski, Vaclav Petřiček, Andrzej Katrusiak

**Affiliations:** a National Centre for Nuclear Research, Andrzeja Sołtana 7, Otwock, Świerk 05-400, Poland; b Institute of Physics; Department of Structure Analysis, Academy of Sciences of the Czech Republic, Cukrovarnicka 10, Prague 6 16253, Czech Republic; c Faculty of Chemistry, Adam Mickiewicz University in Poznań, Umultowska 89 b, Poznań 61-614, Poland; Alfred University, USA

**Keywords:** metal-free perovskite, polymorphism, disorder, ionic crystals

## Abstract

The structure and stoichiometry of a new metal-free and ammonium-free compound [dabcoH_2_]^2+^H_3_O^+^Br^−^_3_ (where [dabcoH_2_]^2+^ = 1,4-di­aza­bicyclo­[2.2.2]octane dication) correspond to the general formula *ABX*_3_ characteristic of perovskites. In enantiomorphic trigonal polymorph α of [dabcoH_2_]^2+^H_3_O^+^Br^−^_3_, the corner-sharing [H_3_O]Br_6_ octahedra combine into a 3D framework embedding [dabcoH_2_]^2+^ dications in pseudo-cubic cages. In the more dense polymorph β, the face-sharing [H_3_O]Br_6_ octahedra form 1D polyanionic columns separated by [dabcoH_2_]^2+^ dications. These different topologies correlate with different crystal fields around the cations and their different disorder types: orientational disorders of [dabcoH_2_]^2+^ dications and H_3_O^+^ cations in polymorph α and positional disorder of [H_3_O]^+^ cations in polymorph β. The orientational disorder increases the lengths of OH⋯Br hydrogen bonds in polymorph α, but NH⋯Br distances of ordered dabcoH_2_ dications are longer in polymorph β. The presence of polar [H_3_O]^+^ cations in [dabcoH_2_]^2+^H_3_O^+^Br^−^_3_ polymorphs offers additional polarizability of the centres compared with analogous metal-free [dabcoH_2_]^2+^[NH_4_]^+^Br^−^_3_ perovskite.

## Abstract 2

A metal-free and ammonium-free perovskite *AB*Br^−^_3_ (where *A* = 1,4-di­aza­bicyclo­[2.2.2]octane dication and *B* = H_3_O^+^) has been obtained in two forms: polymorph α with corner-sharing, and polymorph β with face-sharing octahedra [H_3_O^+^]Br_6_.

## 1. Introduction

Perovskites constitute a wide group of crystalline materials with the characteristic formula *ABX*_3_ and structures built from corner-sharing *BX*_6_ octahedra and *A* cations in the cubic voids. The importance of perovskites is connected with their properties such as ferroelectric and relaxor properties (Strukov & Levanyuk, 1998 ▸), and numerous applications, for example as digital memories (Scott, 2000 ▸), sensors or photovoltaics (Szafrański & Katrusiak, 2017 ▸, 2016 ▸). The mineral CaTiO_3_, discovered in 1839 by Gustav Rose in the Ural mountains was named *perovskite*, after mineralogist Lev Alekseyevich von Perovski. Later, the name *perovskite* was used to describe a wider group of minerals with analogous structures and the general formula *ABO*_3_, then extended to *ABX*_3_, where *X* was a halide anion. Finally, organic–inorganic hybrid perovskites were designed, with large complex unit cells, and which still correspond to the networks built of corner-sharing octahedra or the octahedra corners connected through organic linkers, sometimes of considerable size (Boström & Goodwin, 2021 ▸). Also, 2D and 1D perovskite analogues differentiated in the composition (*e.g. ABO*_4_, CsPb_2_Br_5_), ionicity and topologies (*e.g.* corner/edge/face-sharing octahedra) are also often described as perovskite analogues. Properties and applications of perovskite materials are often connected with their phase transitions and symmetry changes at phase transitions, involving ionic displacements or tilts of the *BX*_6_ octahedra (Glazer, 1972 ▸; Howard & Carpenter, 2010 ▸; Carpenter & Howard, 2009 ▸). They can induce spontaneous polarization and ferroelectricity of crystals, such as for example in BaTiO_3_ and PbTiO_3_ (Megaw, 1946 ▸, 1952 ▸; Shirane *et al.*, 1950 ▸; Shirane & Takeda, 1952 ▸; Shirane & Pepinsky, 1953 ▸; Nelmes & Kuhs, 1985 ▸). In some structures, the symmetry and properties are connected with the disorder of the ions. In recent years, metal-free perovskites are sought for their applications in sensors, detectors, light-emitting diodes (LEDs), photovoltaics and, generally, optoelectronics (Song *et al.*, 2020 ▸; 2021*a* ▸; 2021*b* ▸). The main advantages of organic and hybrid organic–inorganic substitutes of ceramic perovskites are their reduced toxicity, owing to the absence of heavy metals, lower cost of production and processing (formation of thin layers, also in the flexible form) and their easier environment-friendly disposal and recycling. Owing to weaker cohesion forces, bio-friendly metal-free perovskites can exhibit increased sensitivity to external stimuli (Cui *et al.*, 2021 ▸).

Recently, metal-free perovskites involving piperazine, dabco and ammonium cations (NH^4+^) were discovered (Bremner *et al.*, 2002 ▸), and later their analogues [dabcoH]^2+^[NH_4_]^+^Br^−^_3_ and [*M*dabcoH]^2+^[NH_4_]^+^Br^−^_3_ (*M*dabco stands for N-methyl­ated *dabco*, *i.e.* 1,4-di­aza­bicyclo­[2.2.2]octane, C_6_H_12_N_2_) were thoroughly investigated (Ye *et al.*, 2018 ▸; Morita *et al.*, 2020 ▸). These are considered environment-friendly and cheap alternatives to mineral perovskites (Li & Ji, 2018 ▸; Gao *et al.*, 2021 ▸). At present, we report another metal-free perovskite compound [dabcoH_2_]^2+^[H_3_O]^+^Br^−^_3_ obtained in the form of two polymorphs, α and β. Polymorph α has the structure of the analogous metal-free 3D perovskite [dabcoH_2_]^2+^[NH_4_]^+^Br^−^_3_, where the [H_3_O]Br_6_ octahedra share vertices (Ye *et al.*, 2018 ▸). In the structure of polymorph β, [H_3_O]Br_6_ octahedra share faces in 1D columns (Bremner *et al.*, 2002 ▸; Ye *et al.*, 2018 ▸). Both polymorphs α and β of [dabcoH_2_]^2+^[H_3_O]^+^Br^−^_3_ are disordered, but in a different manner. It is characteristic that disorder effects are essential for the properties of many types of crystals, including perovskites and dabco monosalts, where the disorder is connected to the ferroelectric and relaxor properties (Szafrański & Katrusiak, 2004 ▸). Our present study is primarily aimed at identifying the structural features of the [dabcoH_2_]^2+^H_3_O^+^Br^−^_3_ polymorphs.

## 2. Experimental

Single crystals of [dabcoH_2_]^2+^[H_3_O]^+^Br^−^_3_, where [dabcoH_2_]^2+^ of the formula [C_6_H_14_N_2_]^2+^ stands for diprotonated 1,4-di­aza­bicyclo­[2.2.2]octane, were found as a small fraction of crystallizations aimed at growing relaxor ferroelectric dabcoH^+^ bromide (dabcoHBr) from the aqueous solution of dabco and HBr in a 1:1 equimolar ratio (Budzianowski & Katrusiak, 2006 ▸; Szafrański & Katrusiak, 2004 ▸). The X-ray diffraction studies of selected single crystals revealed the presence of a tri-component salt [dabcoH_2_]^2+^ hydro­nium tribromide, [dabcoH_2_]^2+^[H_3_O]^+^Br^−^_3_ (polymorph α). Later, this compound (polymorph β) was obtained close to 100% yield by cooling and slowly evaporating the aqueous solution of dabco with the hydro­bromic acid at a 1:3 molar ratio (the initial crystallization of the equimolar dabco:HBr aqueous solution revealed polymorph α only). The single-crystal X-ray diffraction data (Table 1 ▸) were measured with a KUMA KM4-CCD diffractometer with a graphite-monochromated fine-focus Mo *K*α tube and an Oxford Diffraction XCalibur R diffractometer with a fine-focus X-ray source from a Cu *Kα* tube and Ruby CCD detector; using the latest version of *CrysAlis* and *CrysAlis PRO* software (Rigaku OD, 2003 ▸, 2019*a* ▸). The crystal structure of polymorph α-[dabcoH_2_]^2+^[H_3_O]^+^Br^−^_3_ was partly solved by direct methods in *ShelxS-97* (Sheldrick, 2008 ▸) and then *JANA* (Petříček *et al.*, 2014 ▸) produced the model. Because of the disorder in the structure, we attempted structural refinements in the lower-symmetry space groups with *ShelxL* (Sheldrick, 2015 ▸; Barbour, 2020 ▸; Hübschle *et al.*, 2011 ▸) and *JANA* (Petříček *et al.*, 2014 ▸). Finally, we established that polymorph α-[dabcoH_2_]^2+^[H_3_O]^+^Br^−^_3_ crystallizes in the enantiomorphic trigonal space group *P*3_2_21 (no indication of racemic twinning was detected); the refinement of its structure revealed disorder of the dabcoH_2_ dications in two orientations with nearly equal site-occupation factors of 0.53:0.47(2) (Table 1 ▸). Similar procedures were applied for solving and refining polymorph β-[dabcoH_2_]^2+^[H_3_O]^+^Br^−^_3_ in the trigonal space group *P*3*c*1, where positional disorder was found for two of three symmetry-independent H_3_O^+^ cations; they are disordered at different rates, each in two sites located on a threefold axis (Table 1 ▸). The structure of polymorph β approximates the structure with a 3× smaller unit cell and the symmetry of the space group *P* 62*c* (*cf.* Table S1, Model 4 of the supporting information). The drawings of crystal structures were prepared with the programs *Mercury* (Macrae *et al.*, 2020 ▸), *POV-Ray* (Barbour, 2020 ▸; Cason, 2004 ▸) and *Vesta* (Momma & Izumi, 2011 ▸). Selected structures were presented as autostereograms to facilitate their 3D perception (Katrusiak, 2001 ▸).

The final crystal and structural data and experimental details for both polymorphs are summarized in CIF format in the Cambridge Crystallographic Database Centre as supplementary publications 2132130 and 2132131. They can be obtained free of charge from the Cambridge Structural Database at https://www.ccdc.cam.ac.uk/structures/.

## 3. Discussion

**Figure 1:**
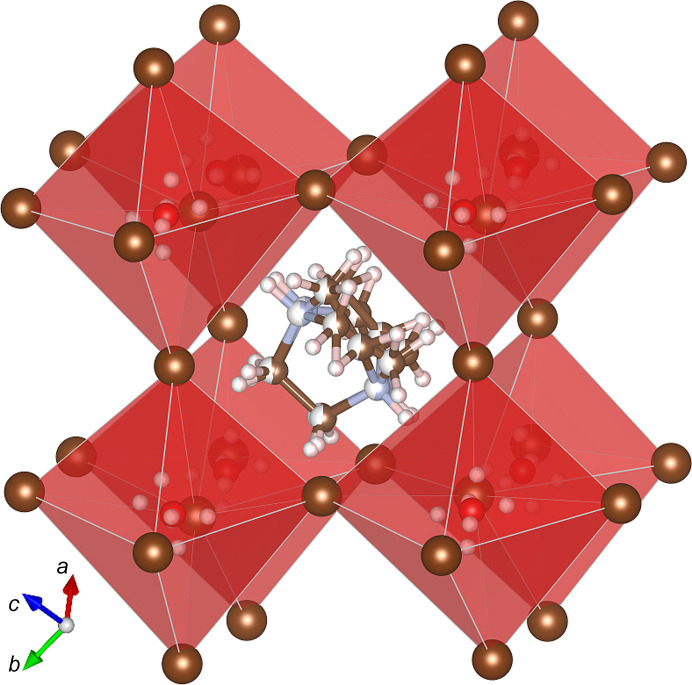
One pseudo-cubic subunit of the trigonal polymorph α-[dabcoH_2_]^2+^[H_3_O]^+^Br^−^_3_, extracted from the 3D network of corner-sharing [H_3_O]Br_6_ octahedra (*cf.* Figs. S4, S5 and S6–S9). Colour and size code: large brown spheres Br, medium blue N, red O, medium brown C, small white H; two colours indicate the partial occupation of disordered N and C atoms.

The structure of polymorph α-[dabcoH_2_]^2+^[H_3_O]^+^Br^−^_3_, where the corner-sharing [H_3_O]Br_6_ octahedra are connected in a 3D framework occluding dabcoH_2_ dications (Fig. 1 ▸) clearly corresponds to the classical perovskite structures of the formula *AB*Br_3_ (Glazer, 1972 ▸; Megaw, 1946 ▸). Moreover, the symmetry of the polymorph α-[dabcoH_2_]^2+^[H_3_O]^+^Br^−^_3_ can be connected to the tilts of the [H_3_O]Br_6_ octahedra, consistent with Glazer’s code *a^-^a^-^a* ^-^ for mineral perovskites. However, due to the non-spherical symmetry of H_3_O^+^ and [dabcoH_2_]^2+^ cations, the unit-cell volume is increased and the crystal symmetry of α-[dabcoH_2_]^2+^[H_3_O]^+^Br^−^_3_ is lowered to one of the enantiomeric space groups *P*3_2_21 or *P*3_1_21. The trigonal unit cell (*Z* = 6) of α-[dabcoH_2_]^2+^[H_3_O]^+^Br^−^_3_ comprises six prototypic perovskite pseudo-rhombohedral sub-units (*Z*′ = 1). An average prototypic pseudo-rhombohedral unit (pR) can be represented in terms of the trigonal (Tr) unit vectors according to the matrix (*cf*. Figs. 1 ▸ and 2 ▸):

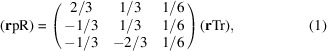

where the vector indices refer to lattices Tr and pR. This transformation yields the idealized prototypic rhombohedral unit cell, of the dimensions *a*_pR_ = 6.753 Å and α_pR_ = 90.40°, close to the average of the true dimensions of the pseudo-rhombohedral cell: *a*_pR_ = 6.720, *b*_pR_ = 6.784, *c*_pR_ = 6.755 Å, α_pR_ = 90.12°, β_pR_ = 90.67° and α_pR_ = 90.42° (*cf*. Figs. 1 ▸ and 2 ▸). The reverse transformation, from the prototypic rhombohedral sub-unit pR to the trigonal unit cell Tr, is

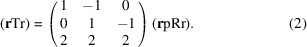

In *ABX*_3_ perovskite structures the interactions between cations (*B*) and anions (*X*) forming the 3D polyanionic framework [*BX*_3_]_*n*_ are mainly electrostatic, like those between the framework and cations *A* contained in the cages. The shortest contacts in the structure of α-[dabcoH_2_]^2+^[H_3_O]^+^Br^−^_3_ confirm that the H_3_O^+^ cations are OH⋯Br^−^ bonded into the 3D framework (Table 2 ▸). The shortest distances C—H⋯Br^−^ are listed in Table 3 ▸. Also, the [dabcoH_2_]^2+^ cations are NH⋯Br^−^ bonded to the linker anions, and these hydrogen bonds are somewhat shorter than those assigned to the framework. Such short contacts between the central cation and the atoms that form the cages are often present in perovskite structures and are considered a significant contribution to the stability and stiffness of the crystals (Ciupa-Litwa *et al.*, 2020 ▸; Collings *et al.*, 2016 ▸; Scatena *et al.*, 2021 ▸; Adjogri & Meyer, 2020 ▸; Hou *et al.*, 2020 ▸).

**Figure 2:**
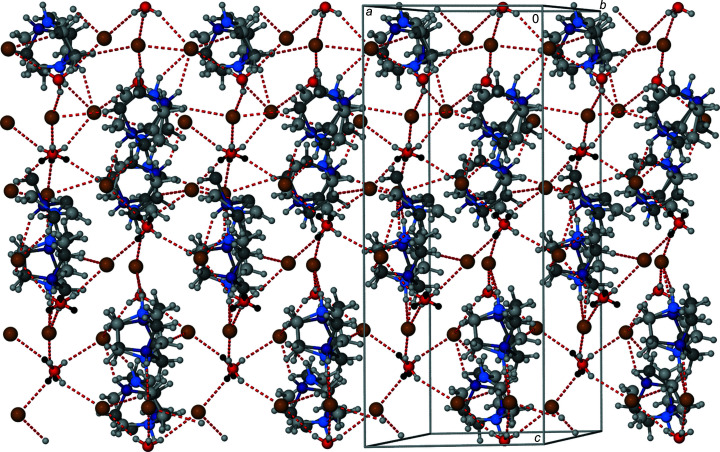
Autostereographic view (Katrusiak, 2001 ▸) of the structure of α-[dabcoH_2_]^2+^H_3_O^+^Br^−^_3_. Hydrogen bonds are indicated by cyan lines. Colour code: brown Br, red O; for disordered cations their different positions are distinguished by two colours: light and dark blue N, light and dark grey C; light-grey and black H.

All hydrogen donors in the structure of α-[dabcoH_2_]^2+^[H_3_O]^+^Br^−^_3_ are disordered. The hydro­nium H_3_O^+^ cations are orientationally disordered in two positions around the oxygen atom. The three hydrogen atoms are located in six half-occupied sites, consistent with the H_3_O^+^ dimensions (Lundren & Olovsson, 1976 ▸), as shown in Figs. 1 ▸, 2 ▸ and S1–S8 (Table S2). Each of the six half-occupied hydrogen sites is involved in one OH⋯Br^−^ hydrogen bond [Figs. S3(*a*) and S3(*b*)]. Dication [dabcoH_2_]^2+^ is disordered in two orientations. The dication is involved in two OH⋯Br^−^ hydrogen bonds. Each of these hydrogen bonds is split between two half-occupied hydrogen sites (Fig. 2 ▸). There are also short distances (∼2.8 to ∼3 Å) between Br ions and methyl­ene hydrogen atoms. All hydrogen bonds listed in Table 2 ▸ can be classified as weak interactions, which are consistent with the considerable disorder of this structure. Like for the mineral perovskites, the symmetry of the crystal field around the cations plays a crucial role in their disorder.

**Figure 3:**
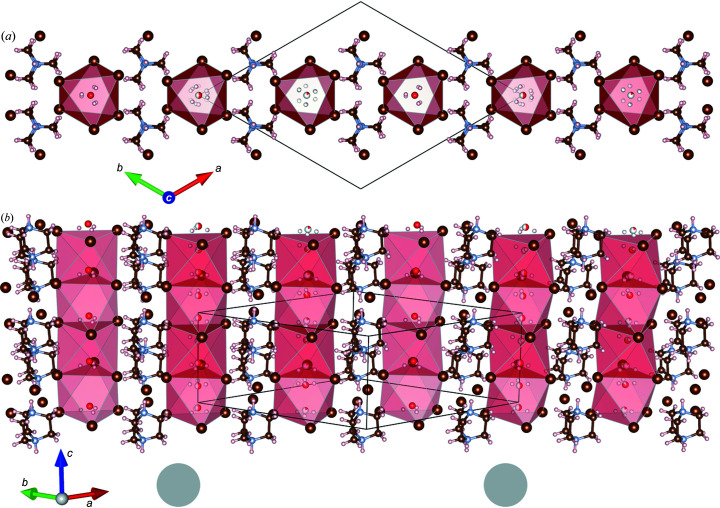
Polymorph β-[dabcoH_2_]^2+^[H_3_O]^+^Br^−^_3_ with chains of face-sharing octahedra: (*a*) parallel view along the [001] direction; and (*b*) autostereographic view (Katrusiak, 2001 ▸) comparing the columns of face-sharing octahedra – the full translational symmetry along the unit-cell diagonal across the page is indicated by two grey circles below the drawing. The colour code for atoms is the same as in Fig. 1 ▸.

**Figure 4:**
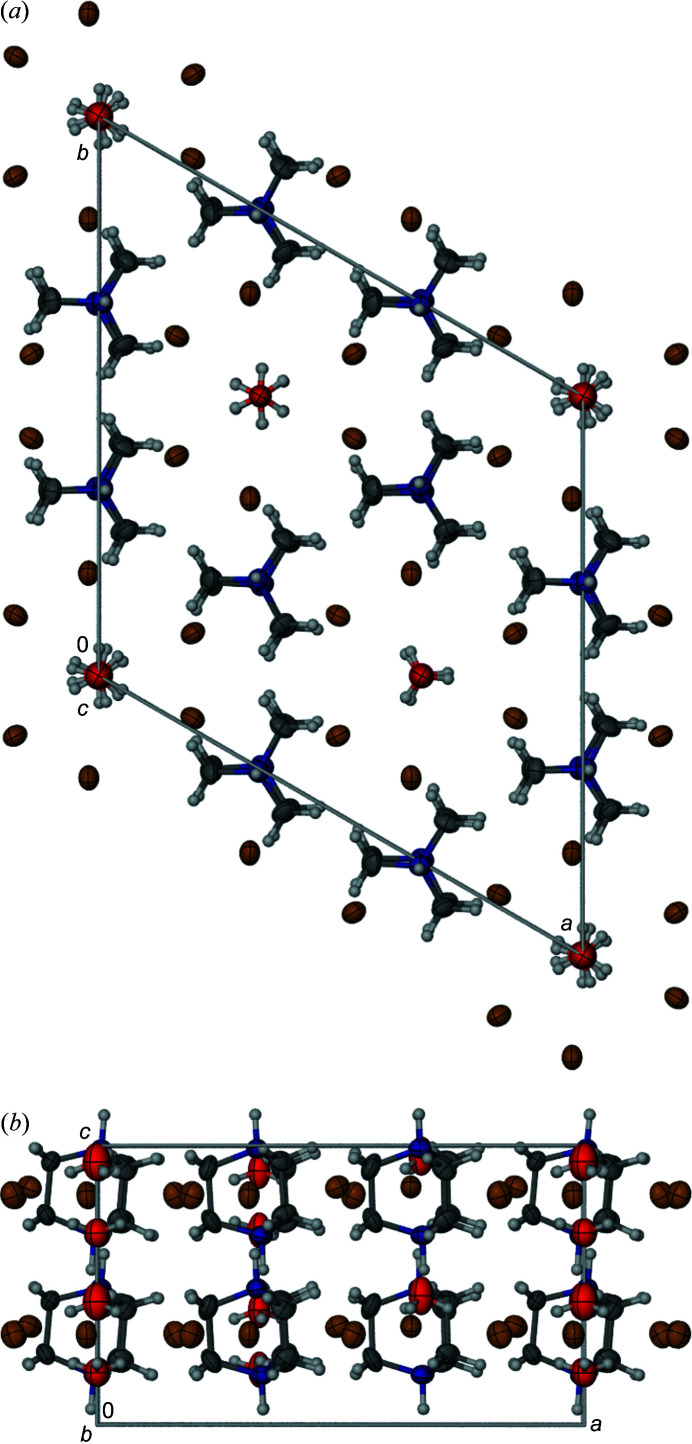
Crystal structure of polymorph β-[dabcoH_2_]^2+^[H_3_O]^+^Br^−^_3_ projected along (*a*) the [001] direction and (*b*) the [010] direction. Partially occupied sites of the disordered hydro­nium cations are indicated. Colour code: brown Br; blue N, red O; dark grey C; light grey H.

In addition to the trigonal polymorph α-[dabcoH_2_]^2+^[H_3_O]^+^Br^−^_3_, we found another concomitant trigonal polymorph β-[dabcoH_2_]^2+^[H_3_O]^+^Br^−^_3_ (Table 1 ▸, Fig. 3 ▸). In its structure, the octahedra of hydro­nium cations and bromine anions ([H_3_O]Br_6_) are face-to-face arranged into columns and the [dabcoH_2_]^2+^ dications are located between these columns. However, in contrast to polymorph α, in polymorph β-[dabcoH_2_]^2+^[H_3_O]^+^Br^−^_3_ the [dabcoH_2_]^2+^ dications are ordered in general positions, while out of three independent hydro­nium cations [H_3_O]^+^ two are disordered. Each of these two [H_3_O]^+^ cations is disordered between a pair of sites along the same column along *z*, as illustrated in Fig. 4 ▸. The distance between the pairs of partially occupied oxygen sites (Table 1 ▸) is approximately equal to 1/4 of the unit-cell parameter *c*, *i.e.* about 2 Å. It is intriguing that the oxygen sites disordered along the threefold axes lie off the centre of the [H_3_O]^+^Br_6_ octahedra, while the centres of the octahedra are at the midpoints between the oxygen sites; the disordered [H_3_O]^+^ cation is also located off its octahedron centre (Fig. 4 ▸). These off-centre sites of the oxygen atoms result in O⋯Br distances (Table 3 ▸) significantly shorter compared with those in α-[dabcoH_2_]^2+^[H_3_O]^+^Br^−^_3_ (Table 2 ▸). The off-centre positional disorder of hydro­nium cations in β-[dabcoH_2_]^2+^[H_3_O]^+^Br^−^_3_ suggests that the OH⋯Br hydrogen bonds favour shorter distances than those between the bromine anion and the octahedron centre. The longer O⋯Br distances in α-[dabcoH_2_]^2+^[H_3_O]^+^Br^−^_3_ can be attributed to the orientational dynamic disorder of the H_3_O^+^ cation; its hydrogen atoms share the tetrahedrally located sites with the lone-electron pair: the tetrahedral sites do not match the octahedral locations of the bromine anions around, while the lone-electron pair does not contribute to the attraction of the hydrogen bonds, but it can be associated with some repulsion. On the other hand, the [dabcoH_2_]^2+^ dications in β-[dabcoH_2_]^2+^[H_3_O]^+^Br^−^_3_ are stabilized in the ordered position by their trigonal environment and form NH⋯Br^−^ contacts that are significantly longer than those of disordered [dabcoH_2_]^2+^ dications in the pseudo-cubic crystal environment of α-[dabcoH_2_]^2+^[H_3_O]^+^Br^−^_3_ (Tables 2 ▸ and 3 ▸).

The crystallographic information about polymorphs explains the origin of different disorder types in their structure. The [dabcoH_2_]^2+^ dications can assume either the *D*_3_ twisted left or right propeller conformation, or the averaged *D*_3h_ symmetry (Olejniczak *et al.*, 2013 ▸). However, in polymorph α the dications are located in the pseudo-cubic cages, approximating the *O*_h_ symmetry. Consequently, the crystal environment of polymorph α does not stabilize one specific orientation of the cation. On the other hand, the trigonal symmetry of polymorph β results in the trigonal surrounding of the dications, matching their symmetry well. Indeed, the shape of pseudo-*D*_3h_-symmetric dications is consistent with the trigonal crystal environment. In polymorph α, the hydro­nium cations are located at the vertices of the pseudo-cubic cages, which does not favour any of their displacements. In polymorph β, the [H_3_O]^+^ cations are located in the channel-like surrounding of the threefold axes, which does not appear to strongly favour any site along *z* and results in the disorder of the cations. Interestingly, the structure of polymorph β approximates the higher symmetry of space group *P* 62*c*, with the unit cell 3× smaller (Figs. S9–S16, Tables S1 and S2). This high symmetry is broken by small tilts of the dications and displacements of the Br anions, as well as by the differences between the hydro­nium cations, ordered and disordered to different extents (Table 1 ▸), as shown in the structure projections in Fig. 4 ▸.

## 4. Conclusions

Polymorphs of [dabcoH_2_]^2+^[H_3_O]^+^Br^−^_3_ illustrate the universality of perovskite structures and their characteristic features. The general formula *ABX*_3_ of perovskites, initially associated with minerals and ionic crystals, can clearly be extended to various hybrid organic–inorganic compounds, where organic central *A* cations interact with (in)organic *X* linkers, binding the *B* metal centres in the systems with much weaker cohesion forces, such as hydrogen bonds and electrostatic interactions between molecular ions. Even the metal-free compounds still display the characteristic structural properties of perovskites, controlled by the tilts of *BX*_6_ octahedra and disorder of the cations: the size and orientation of the molecular ions are additional factors responsible for the crystal symmetry and macroscopic properties of the hybrid and metal-free perovskites. The structures of both polymorphs of [dabcoH_2_]^2+^[H_3_O]^+^Br^−^_3_ are disordered under normal conditions, which is an indication of possible temperature and pressure-induced phase transitions of properties. Both orientational and positional disorder are present and they interplay with the crystal field around the cations and their hydrogen bond capabilities. Polymorph α-[dabcoH_2_]^2+^[H_3_O]^+^Br^−^_3_, like its close analogue [dabcoH_2_]^2+^[NH_4_]^+^Br^−^_3_, is one of very few enantiomorphic and polar perovskites reported so far. With the exception of C_4_H_14_N_2_RbCl_3_ (Paton & Harrison, 2010 ▸), the previously reported enantiomorphic perovskites employed chiral cations, for example in (*R*)-, (*S*)-3-(fluoro­pyrrolidinium)MnBr_3_ and in (*R*)-, (*S*)-N,N-di­methyl-3-fluoro­pyrrolidinium CdCl_3_ (Gao *et al.*, 2020 ▸; Peng *et al.*, 2021 ▸), where the enantiomorphic form of the crystal was permanently connected with the chiral cation used for the synthesis. To our knowledge, the polymorphs [dabcoH_2_]^2+^[NH_4_]^+^Br^−^_3_ and α-[dabcoH_2_]^2+^[H_3_O]^+^Br^−^_3_ are the first metal-free enantiomorphic and polar 3D perovskite structures where no permanent chiral cations are present and therefore this structure can be switched between two enantiomorphs. The structure of polymorph β-[dabcoH_2_]^2+^[H_3_O]^+^Br^−^_3_ is polar, which can also potentially result in ferroelectric properties. Thus, in both polymorphs, the substitution of the non-polar [NH_4_]^+^ cation with the polar [H_3_O]^+^ cation can result in increased polarizability of the system and advantageous switchable properties desired for practical applications in optoelectronic devices.

## 5. Related literature

The following references are cited in the supporting information: Rigaku OD (2019*b* ▸, 2020 ▸, 2021 ▸); Clark & Reid (1995 ▸); Laetsch & Downs (2006 ▸).

## Supplementary Material

Crystal structure: contains datablock(s) dhbr1af_41001, dabco1_h168b6_abs2. DOI: 10.1107/S2052252522006406/ct5016sup1.cif

Supporting figures and tables. DOI: 10.1107/S2052252522006406/ct5016sup2.pdf

CCDC references: 2132130, 2132131

## Figures and Tables

**Table 1:** Crystal data and experimental details for [dabcoH_2_]^2+^[H_3_O]^+^Br^−^_3_ polymorphs α and β determined at 297 K and 0.1 MPa Note the different rates of disorder of independent hydro­nium cations in polymorph β.

	α-polymorph	β-polymorph
Chemical formula moiety	[C_6_H_14_N_2_]·3Br^+^·H^−^O^+^	[C_6_H_14_N_2_]·3Br^+^·H^−^O^+^
Disordered cation/ratio	dabcoH^2+^ 0.53:0.47(2) orientation, H_3_O^+^ disordered in 2 half-occupied orientations	2H_3_O^+^ cations positionally disordered, each in 2 sites, 0.51:0.49(3), 0.84:0.16(1)
Empirical formula	C_6_H_17_Br_3_N_2_O	C_6_H_17_Br_3_N_2_O
Formula weight	372.94	372.94
Crystal system	Trigonal	Trigonal
Space group (No.)	*P*3_2_21 (154)	*P*3*c*1 (158)
*a*, *b*, *c* (Å)	9.5838(3), 9.5838(3), 23.2270(8)	16.0425(1), 16.0425(1), 7.9666(7)
α, β, γ (°)	90, 90, 120	90, 90, 120
Volume (Å^3^)	1847.56(13)	1775.61(16)
*Z*/*Z*′	6/1	6/(3 × 1/3)
*V*/*Z*	307.93(2)	295.94(3)
*D_x_* (g cm^−3^)	2.011	2.093
Wavelength (Å)	0.71073	1.54184
θ range (°)	2.454–29.253	3.181–71.234
Min/max indices *h*, *k*, *l*	−13/12, −9/12, −31/30	−19/19, −19/19, −9/9
*F*(000)	1080	1080
Reflections (all)	14765	37462
Independent reflections/*R*_int_	3159/0.0526	2269/0.0573
θ to 100% completeness (°)	25.242	67.684
Max/min transmission	0.985/0.956	0.334/0.060
Data/restraints/parameters	3159/84/201	2269/13/131
GooF on *F* ^2^	1.077	1.053
Final *R*_1_/*wR*_2_ Σ[*I* > 2σ*I*]	0.0415/0.652	0.0287/0.0782
*R*_1_/*wR*_2_ (all data)	0.0705/0.740	0.0319/0.0814
Extinction coefficient	–	0.00143(13)
Absorption coefficient (mm^−1^)	9.792	12.368
Absolute structure parameter	0.005(15)	−0.02(2)
Max diff. peak/hole [eÅ^−3^]	0.548/−0.607	0.443/−0.468

**Table 2:** Hydrogen-bond contacts in α-[dabcoH_2_]^2+^[H_3_O]^+^Br^−^_3_, with the donor–acceptor (*D*⋯*A*) distances shorter than the sum of their van der Waals radii (Bondi, 1964 ▸) and angle *D*—H⋯*A* larger than 110°

*D*—H⋯*A*	*D*—H (Å)	H⋯*A* (Å)	*D*⋯*A* (Å)	*D*—H⋯*A* (°)
O(2w)—H(21w)⋯Br(1A)^i^	0.8201(14)	2.564(16)	3.370(5)	168(6)
O(2w)—H(22w)⋯Br(2A)^ii^	0.8201(14)	2.62(2)	3.394(4)	158(6)
O(2w)—H(23w)⋯Br(3A)^iii^	0.8201(15)	2.625(16)	3.4303(12)	167(6)
O(1w)—H(11w)⋯Br(3A)^iv^	0.8201(14)	2.62(2)	3.411(5)	162(6)
O(1w)—H(12w)⋯Br(1A)	0.8201(14)	2.59(2)	3.3591(9)	156(5)
O(1w)—H(13w)⋯Br(2A)^v^	0.8201(15)	2.639(19)	3.439(5)	166(6)
N(1A)—H(1A)⋯Br(2A)	0.98	2.39	3.244(13)	145.0
N(2A)—H(2A)⋯Br(3A)	0.98	2.37	3.240(14)	148.1
N(1B)—H(1B)⋯Br(2A)	0.98	2.36	3.249(15)	150.1
N(2B)—H(2B)⋯Br(3A)	0.98	2.36	3.243(16)	149.0

Symmetry codes: (i) 1 − *x*, *y* − *x*, 2/3 − *z*; (ii) *x* −*y* + 1, −*y* + 1, 1/3 − *z*; (iii) 2 − *x*, 1 − *x* + *y*, 2/3 − *z*; (iv) *y*, *x*, 1 − *z*; (v) −*x*, *y* − *x*, 2/3 − *z*.

**Table 3:** Shortest interionic contacts corresponding to hydrogen bonds NH⋯Br and OH⋯Br in β-[dabcoH_2_]^2+^[H_3_O]^+^Br^−^_3_; *cf*. Table 2 ▸

*D*—H⋯*A*	*D*—H (Å)	H⋯*A* (Å)	*D*⋯*A* (Å)	*D*—H⋯*A* (°)
O(1w)—H(1w)⋯Br(1)	0.82(2)	2.31(3)	3.121(4)	169(12)
O(2w)—H(2w)⋯Br(2)	0.83(2)	2.6(2)	3.102(8)	119(18)
O(1wA)—H(1wA)⋯Br(1)	0.83(2)	2.23(11)	3.037(15)	165(40)
O(2wA)—H(2wA)⋯Br(2)	0.83(2)	2.33(4)	3.148(6)	171(17)
O(3w)—H(3w)⋯Br(3)	0.84(3)	2.30(5)	3.079(4)	154(9)
N(1)—H(1)⋯Br(1)	0.98	2.82	3.541(6)	130.7
N(1)—H(1)⋯Br(2)	0.98	2.96	3.602(7)	124.2
N(1)—H(1)⋯Br(3)	0.98	3.22	3.837(5)	122.2
N(2)—H(2)⋯Br(1)^i^	0.98	3.18	3.787(6)	121.6
N(2)—H(2)⋯Br(2)^i^	0.98	3.06	3.734(7)	126.8
N(2)—H(2)⋯Br(3)^i^	0.98	2.79	3.497(5)	129.5
C(1)—H(1A)⋯Br(2)	0.97	2.97	3.598(6)	123.4
C(1)—H(1B)⋯Br(1)^ii^	0.97	2.95	3.738(7)	139.4
C(2)—H(2A)⋯Br(1)	0.97	3.00	3.521(7)	115.4
C(2)—H(2B)⋯Br(3)^iii^	0.97	3.08	3.817(7)	133.9
C(3)—H(3A)⋯Br(3)	0.97	2.96	3.635(6)	127.5
C(3)—H(3B)⋯Br(2)^iv^	0.97	3.12	3.840(7)	132.0
C(4)—H(4A)⋯Br(1)^ii^	0.97	3.12	3.838(7)	131.8
C(4)—H(4B)⋯Br(2)^i^	0.97	3.00	3.593(6)	120.9
C(5)—H(5A)⋯Br(3)^iii^	0.97	3.01	3.781(7)	137.1
C(5)—H(5B)⋯Br(1)^i^	0.97	2.98	3.665(6)	129.0
C(6)—H(6A)⋯Br(2)^iv^	0.97	2.97	3.751(6)	138.8
C(6)—H(6B)⋯Br(3)^i^	0.97	3.00	3.553(6)	117.2

Symmetry codes: (i) *y*, *x*, *z* − 1; (ii) *x*, *x* − *y* + 1, *z* − 1/2; (iii) 1 − *y*, 1 − *x*, *z* − 1/2; (iv) 1 − *x* + *y*, *y*, *z* − 1/2.
