# Supplementary material for: Metal-free enantiomorphic perovskite [dabcoH2]2+[H3O]+Br− 3 and its one-dimensional polar polymorph
Source: IUCrJ. 2022 Jul 16;9(Pt 5):544–50. doi: 10.1107/S2052252522006406 (PMC9438492; doi:10.1107/S2052252522006406)
Supplement: Supplementary file 2 [file m-09-00544-sup2.pdf]

IUCrJ

Volume 9 (2022)

Supporting information for article:

Metal-free enantiomorphic perovskite [dabcoH2]$^{2+}$[H3O]$^{+}$ Br ̄3 and its 1-dimensional polar polymorph

Armand Budzianowski, Vaclav Petříček and Andrzej Katrusiak

S1. α -Polymorph of $[dabcoH_2]^{2+}H_3O^+ Br_3^-$

The structure was solved and refined in space group *P*3₂21 with the $[C_6H_{14}N_2]^{2+}$ cation disordered. Because of the relatively high absorption of the crystal, we calculated the analytical absorption correction (Rigaku Oxford Diffraction, 2019a; Clark, & Reid, 1995) for intensities of reflections. In the final model, all non-H atoms were refined with anisotropic temperature factors and H atoms were located from the molecular geometry with one common isotropic temperature factor. For the hydronium cations, the difference-Fourier map peaks around the oxygen atom indicated a disorder of the cation in two orientations, as shown in Figures S1, S2, S3 and S4a. In the final refinement, the dimensions of each set of H-atoms for one orientation of the H_3O^+ cation were restrained by instructions DFIX 0.82Å for O-H bonds and DANG 1.33Å for non-bonding H···H distances (Table 2).

The twinned crystal structure in space group $P3_2$ [twinning-habit matrix (010 100 00-1)] was also tested. The occupation of twins refined to 0.5, however, this model was unstable during refinement and yielded higher *R* factors.

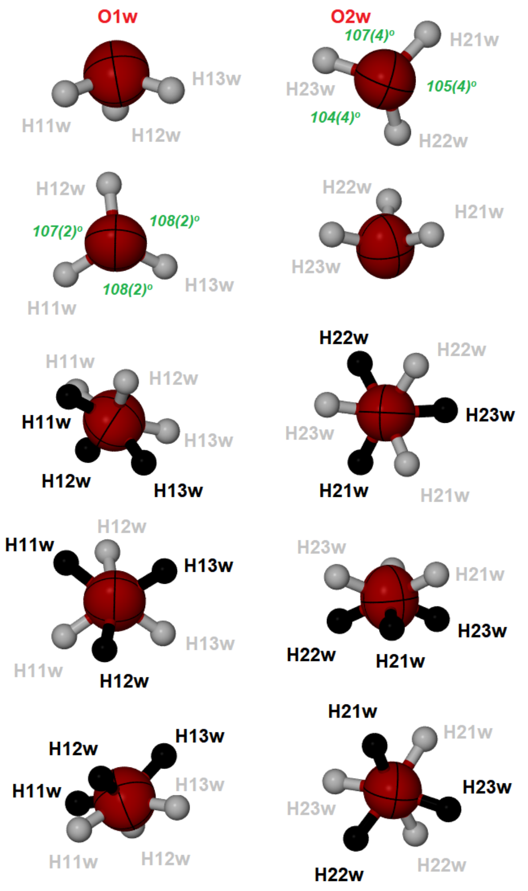

Figure S1 Polymorph α-$[dabcoH_2]^{2+}[H_3O]^+Br_3^-$: two independent disordered hydronium cations $H_3O(1w)^+$ and $H_3O(2w)^+$, located on a 2-fold axis and an inversion centre, respectively. One of two 50% occupied sites (two top views) and both of two disordered sites (three bottom views) are shown in the same projection for each row of drawings. Colour code: grey/black 50%-disordered H-atoms, red O-atoms (100% occupancy).

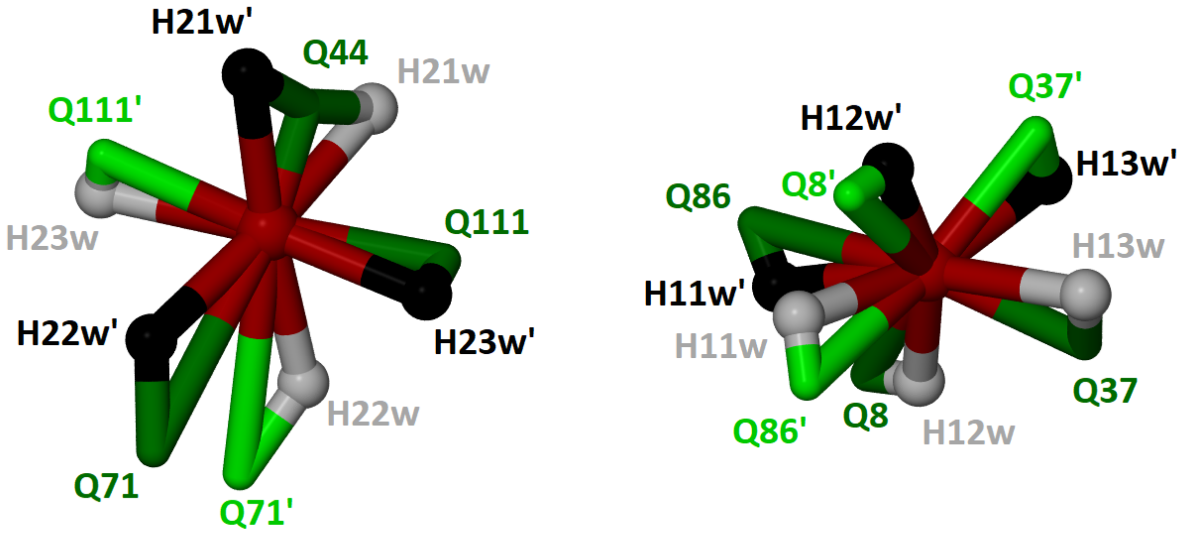

Peak	Peak e-density (e/Å^3)	Distance (Å) to:		Distance (Å) to:	
**Q86**	0.21	0.953	O(1w)	0.311	H(11w)’
**Q8**	0.36	1.011	O(1w)	0.312	H(12w)
**Q37**	0.26	0.796	O(1w)	0.471	H(13w)
**Q44**	0.26	0.592	O(2w)	0.489	H(21w)
**Q71**	0.22	1.192	O(2w)	0.508	H(22w)’
**Q111**	0.20	0.826	O(2w)	0.237	H(23w)’

Figure S2 Location and height of relevant peaks present in the difference Fourier near two independent disordered hydronium cations $H_3O(1w)^+$ and $H_3O(2w)^+$ in polymorph α-[dabcoH$_2$]$^{2+}$[H$_3$O]$^+$Br$^-_3$. Peak Q44 appeared in a special position between two H-atom sites. The partly occupied H sites were located according to these ΔF peaks, following the procedure described above and the chemical information: expected geometry around the oxygen atom and interactions with the bromine atoms. The determination of the H-sites from the ΔF maps and then their constrained refinement lead to the expected structure relative to the oxygen and bromine atoms, as well as to the expected interionic contacts. Distances to peaks were calculated without estimating the standard deviations.

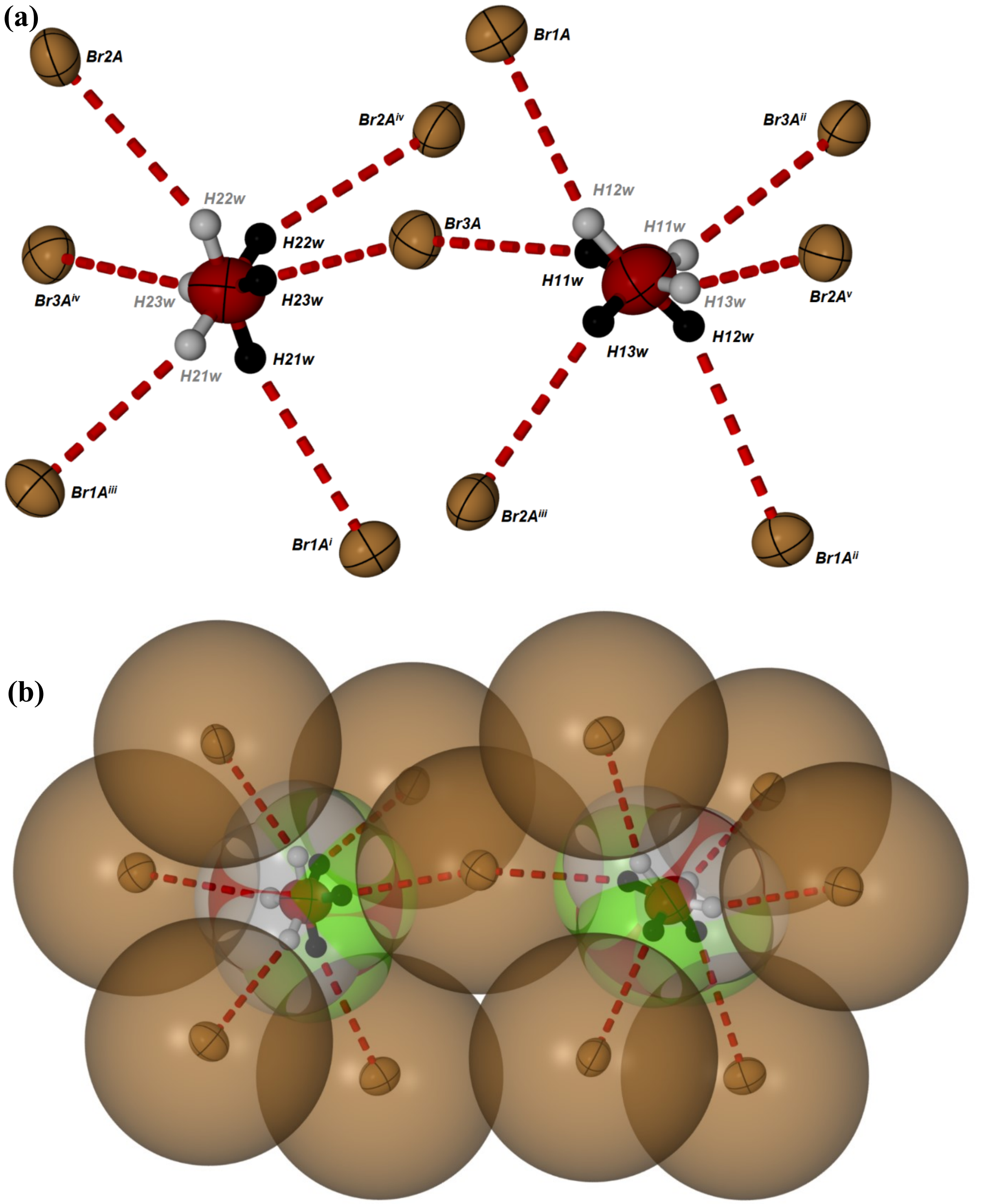

Figure S3 Hydrogen bonds Br···H-O in the polymorph α-$[dabcoH_2]^{2+}[H_3O]^{+}Br_3^{-}$: (a) involving the 50:50 disordered hydronium ions (their orientations distinguished by white and black sites of H-atoms. Symmetry codes: (i) 1+*x*, *y*, *z*; (ii) *y*, *x*, 1-*z*; (iii) 1-*x*, *y*-*x*, 2/3-*z*; (iv) 2-*x*, 1-*x*+*y*, 2/3-*z*; (v) *y*-*x*, 1-*x*, 1/3+*z*. Colour code: Br brown, H grey/black, O red; (b) the same structural fragment with van der Walls spheres added.

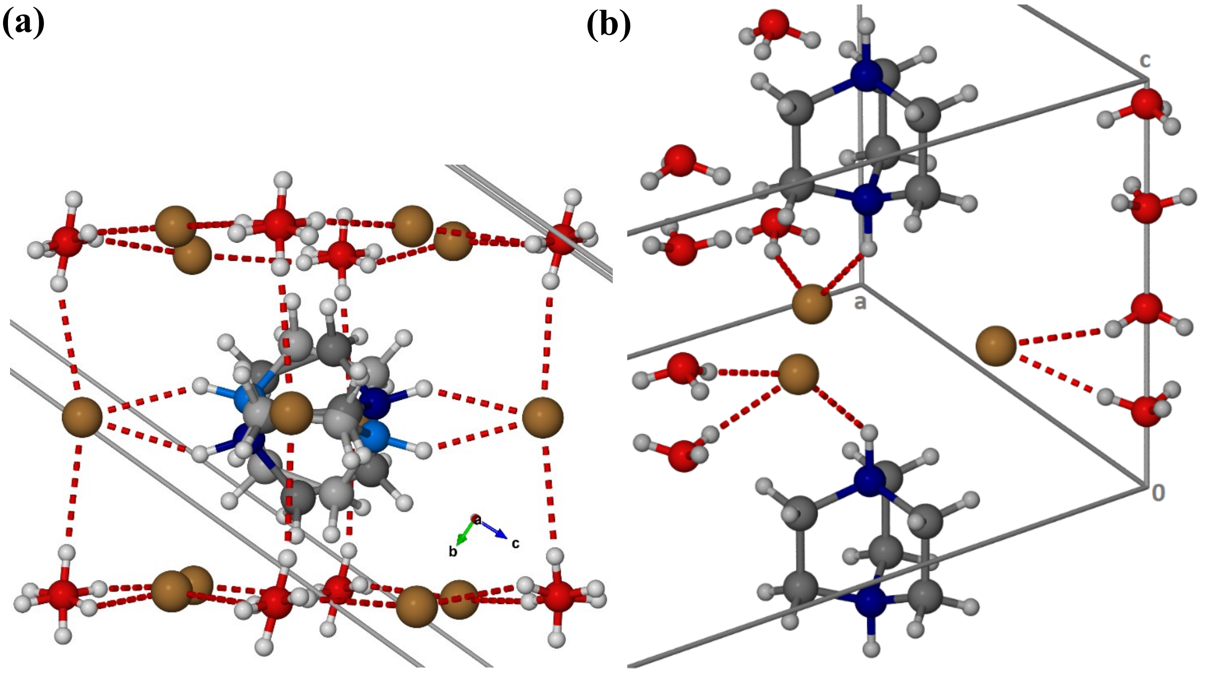

Figure S4 Disordered parts of $[dabcoH_2]^{2+}H_3O^+\ Br_3^-$ polymorphs: (a) two equally occupied (site-occupancy factor SOF=0.5) orientations of $[dabcoH_2]^{2+}$ in H-bonded Br···H-dabco-H···Br in polymorph α; and (b) chains of partly occupied hydronium cations in polymorph β. Hydrogen bonds are indicated by red dotted lines. The long grey lines are the unit-cell edges along *c*. Colours code: brown Br; light / dark blue N, red O; light/dark grey C; light-grey for H; in (a) in polymorph α two colours indicate the partial occupancy of the same site by disordered N and C atoms of dabco dications; H atoms in hydronium cations are located in half-occupied sites (See Figs. S1, S2, S3 and Table S2); (b) in polymorph β all H_3O^+ cations (including the O-atoms) are located in half-occupied sites.

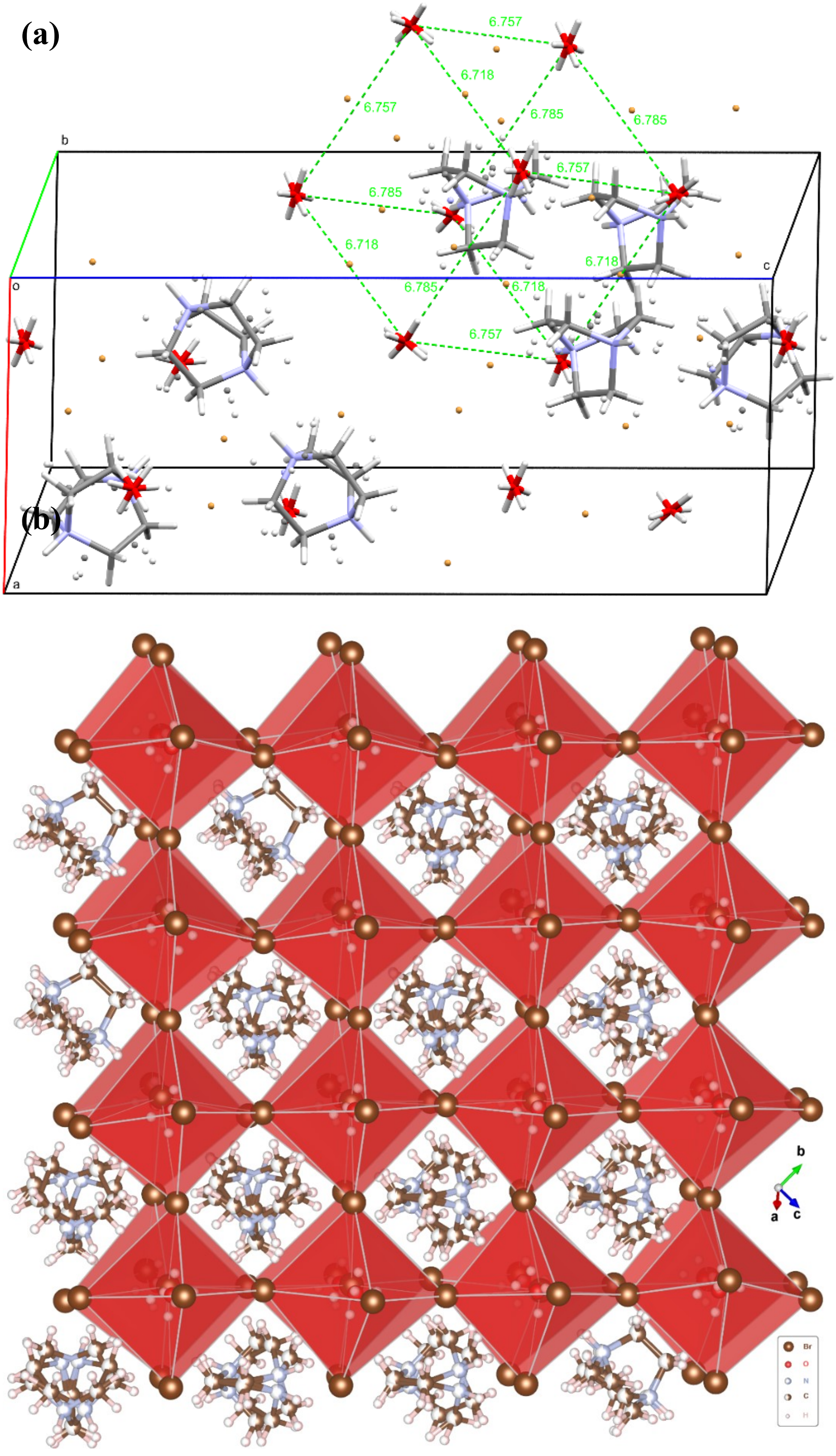

Figure S5 (a) The trigonal unit cell of α-$[dabcoH_2]^{2+}H_3O^+ Br_3^-$ viewed perpendicular to axis [z], with the prototypic perovskite cell edges marked in green. Their dimensions [Å] are measured between the hydronium O-atoms. (b) This structure shown as the network of corner-sharing octahedra (*cf*. Figs. S6-S9). Disordered half-occupied positions of cations $dabcoH_2]^{2+}$ and H_3O^+ are displayed. The colour code for atoms in Fig S4a is: brown Br, blue N, red O, dark grey C, light grey H; in Fig 3b the colour code is: brown Br, blue N, red O, brown small balls C, white H.

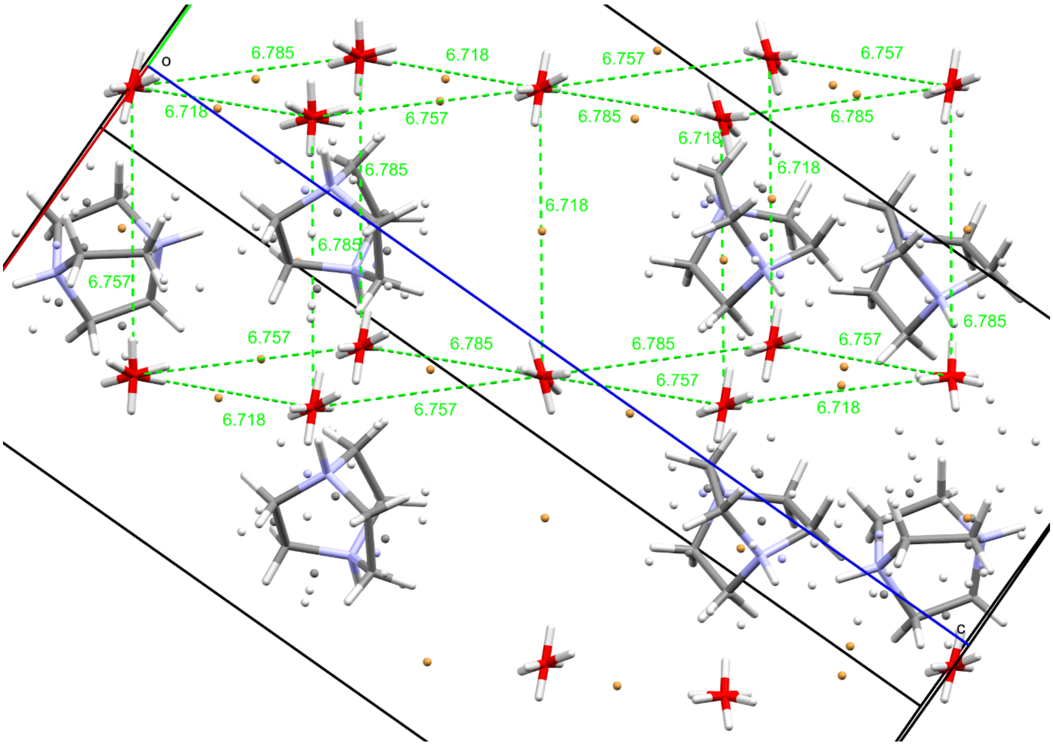

Figure S6 (*Cf.* Figure S5a) – another choice of pseudo-cubic units (green dashed lines between hydronium cations; green numbers are their lengths [Å]) in the structure of α-[dabcoH$_2$]$^{2+}$H$_3$O$^+$ Br$_3^-$ viewed approximately along [110]. The colour code: brown Br; blue N; red O; dark grey C; white H.

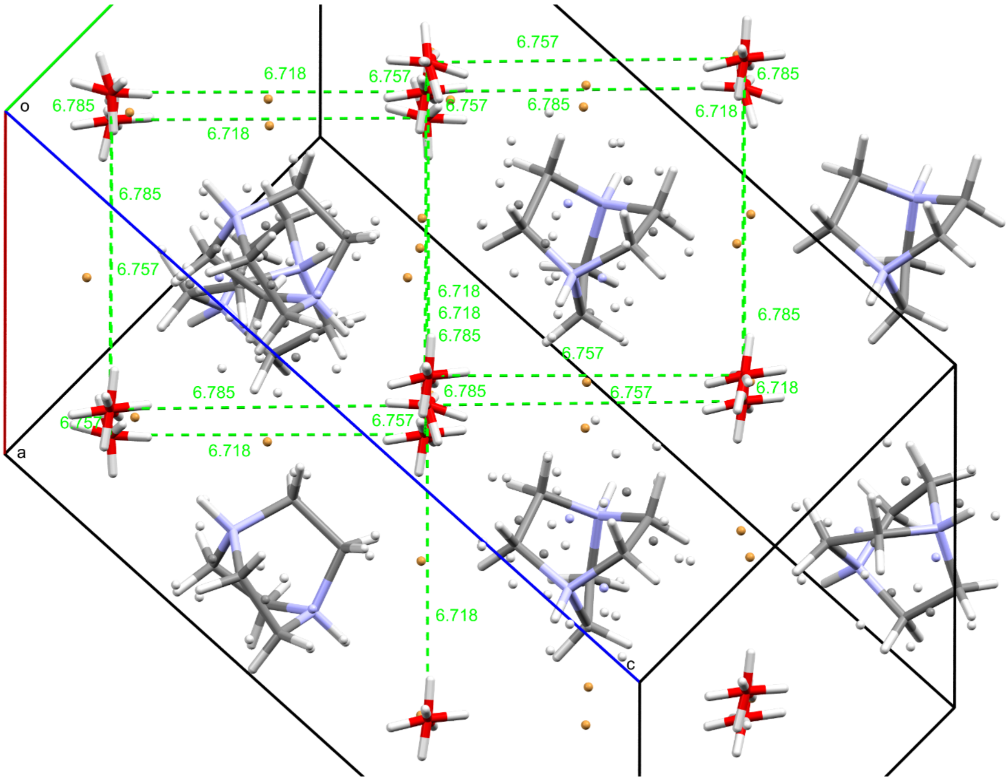

Figure S7 (*Cf.* Figure S6) – another view of pseudo-cubic units in the crystal structure of α-[dabcoH$_2$]$^{2+}$[H$_3$O]$^+$Br$_3^-$. The colour code for atoms: brown Br; blue N; red O; dark grey C; light grey H.

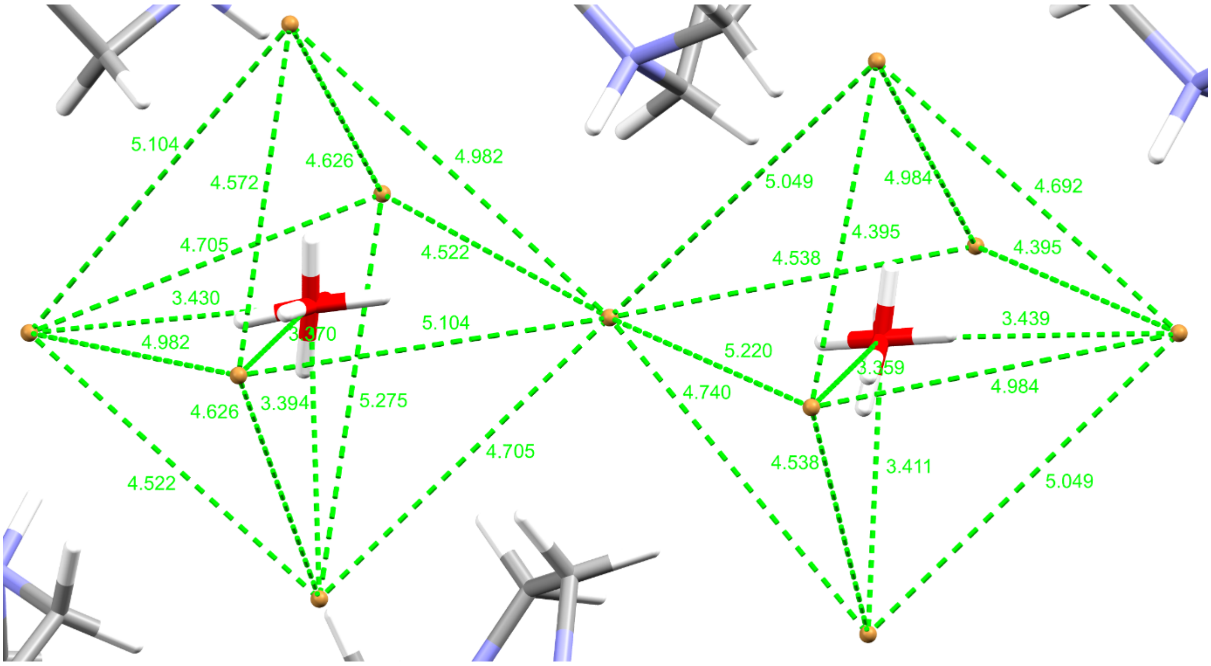

Figure S8 Crystal structure of polymorph α-$[dabcoH_2]^{2+}[H_3O]^{+}Br_3^{-}$ with indicated dimensions of the octahedron edges (Br···Br), as well as contacts Br···O involving the hydronium cation. The colour code for atoms: brown Br; blue N; red O; dark grey C; white H.

S2. β-Polymorph of $[dabcoH_2]^{2+}H_3O^{+}$ Br_3^{-}

Polymorph β was obtained with the 100% yield from the mixture of bromine acid with dabco and water (ratio 3:1 mol.), as confirmed by the powder X-ray diffraction (Fig. S16). Single-crystal X-ray data were measured on the XCaliburR 4-circle diffractometer equipped with a CCD detector and a copper X-ray tube. The CrysAlisPro software was used for collecting the data (Rigaku Oxford Diffraction, 2019b) and its preliminary reduction (Rigaku Oxford Diffraction, 2020). The experimental and crystallographic details of this trigonal structure are listed in Table S1, together with the information for other models tried for investigating the disorder of the hydronium cations and the pseudo-hexagonal model with 3-times smaller unit cell (Figs. S10, S11). The crystal structure was solved straightforwardly by dual methods in ShelxT (Sheldrick, *2015*). The inspection (Rigaku Oxford Diffraction, 2020) of the diffraction layers revealed the hexagonal pseudo-symmetry (Figs. S12-S15). Several best structural models are summarized in Table S1 in order to illustrate the pseudo-symmetry and the disorder of the hydronium cations. The best consistence with the experiment was obtained for the structure of space group *P*3*c*1, with partially disordered H_3O^{+}; a=b=16.04250(10) Å, c=7.9666(7) Å V/Z=295.94(3) Å^3.

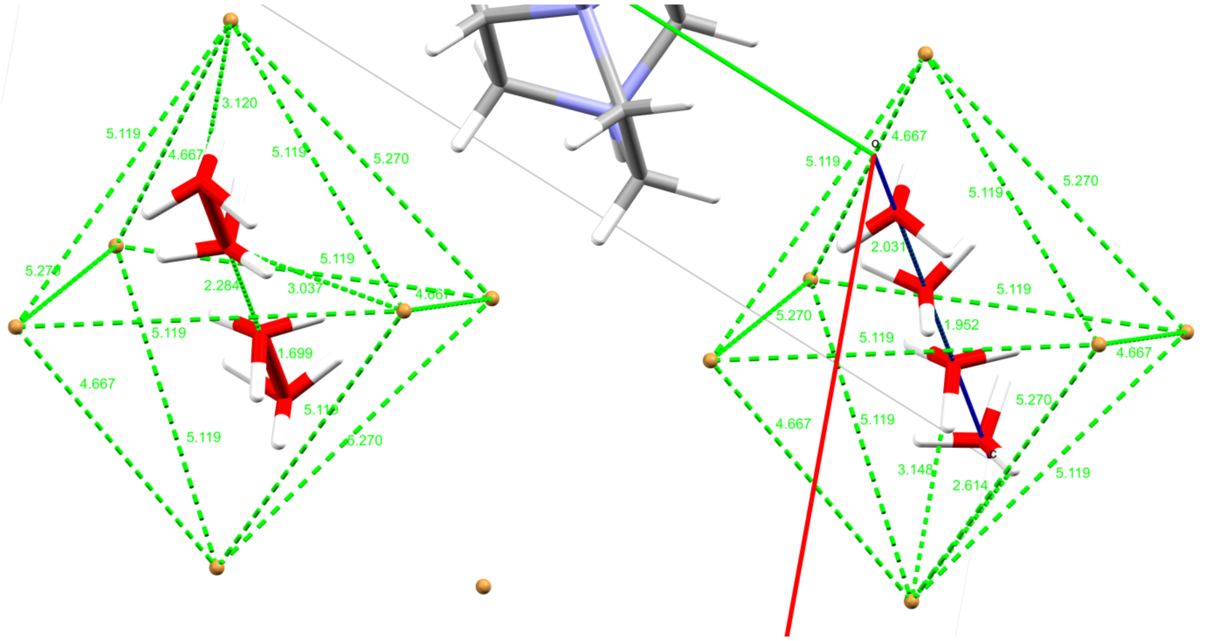

Figure S9 Crystal structure of polymorph β-$[dabcoH_2]^{2+}[H_3O]^{+}Br^{-}_3$ with indicated distances along the octahedron edges (Br···Br), as well as Br···O and O···O between the disordered sites of the hydronium cation. Colour code: brown Br-atoms; blue N; red O; dark grey C; white H. The red, green and blue solid lines from point [000] represent unit cell edges *a*, *b* and *c*, respectively.

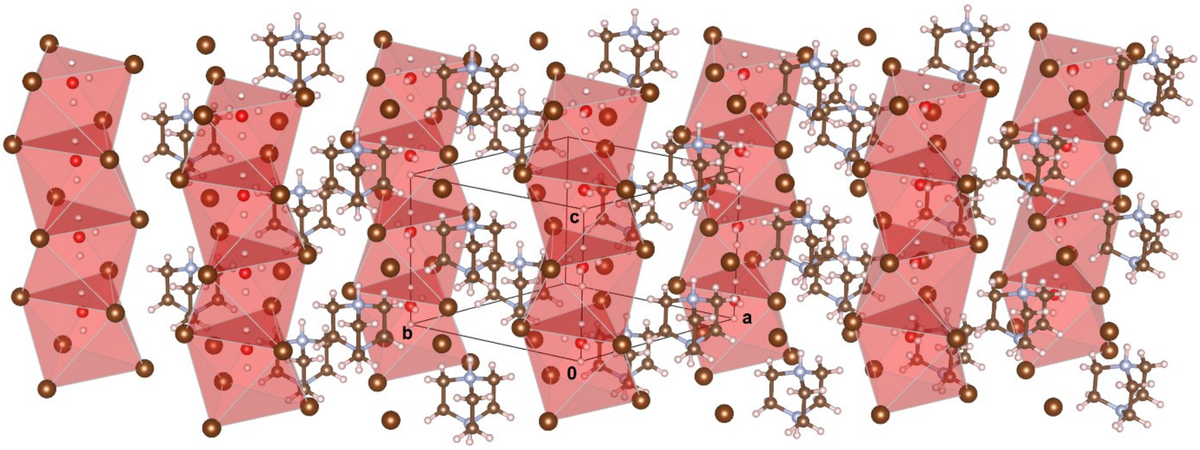

Figure S10 Autostereogram (Katrusiak, 2011) of the pseudo-hexagonal Model 4 (Table S1) of β-$[dabcoH_2]^{2+}[H_3O]^{+}Br^{-}_3$ with indicated chains of face-sharing octahedra (Momma & Izumi, 2011). This structure is similar to $C_6N_2H_{14}{\cdot}NH_4Cl_3$ (Bremner *et al.*, 2002). The colour code for atoms is: brown Br, blue N, red O, brown small balls C, white H.

Table S1 Crystallographic data for β-[dabcoH$_2$]$^{2+}$[H$_3$O]$^+$Br$^-_3$ compared with other models (2-4) tested for this structure.

	**β-polymorph**	Model 2	Model 3	Model 4
Code	**dabco1_h168b6_abs2**	dabco1_h168b3_abs2	dabco1_P-62c	d1wt
Chemical formula moiety	**[C$_6$H$_{14}$N$_2$]$^{2+}$·3Br$^-$ ·H$_3$O$^+$**	[C$_6$H$_{14}$N$_2$]$^{2+}$·3Br$^-$ ·H$_3$O$^+$	[C$_6$H$_{14}$N$_2$]$^{2+}$·3Br$^-$ ·H$_3$O$^+$	[C$_6$H$_{14}$N$_2$]$^{2+}$·3Br$^-$ ·H$_3$O$^+$
Disordered units/ratio	**H$_3$O$^+$ 1:0(0)**<br>**H$_3$O$^+$ 0.51:0.49(3)**<br>**H$_3$O$^+$ 0.84:0.16(1)**	No disorder (constrain to flat H$_3$O$^+$)	No disorder	H$_3$O$^+$ 50:50
Empirical formula	**C$_6$ H$_{17}$ Br$_3$ N$_2$ O**	C$_6$ H$_{17}$ Br$_3$ N$_2$ O	C$_6$ H$_{17}$ Br$_3$ N$_2$ O	C$_6$ H$_{17}$ Br$_3$ N$_2$ O
Formula weight	**372.94**	372.94	372.94	372.95
Crystal system	**trigonal**	trigonal	hexagonal	hexagonal
Space group (No.)	***P*3*c*1 (158)**	*P*3*c*1 (158)	$P\bar{6}2c$ (190)	$P\bar{6}2c$ (190)
Unit cell:<br>*a* [Å]<br>*c* [Å]	 **16.04250(10)**<br>**7.9666(7)**	 16.04250(10)<br>7.9666(7)	 16.04250(10)<br>7.9666(7)	 9.26267(9)<br>7.96755(10)
Volume [Å^3]	**1775.61(16)**	1775.61(16)	1775.61(16)	592.008(13)
Z / *Z'*	**6 / 1**	6 / 1	6 / ½	2 / ⅙
V / *Z*	**295.94(3)**	295.94(3)	295.94(3)	296.004(7)
D_x [g/cm^3]	**2.093**	2.093	2.093	2.092
Wavelength [Å]	**1.54184**	1.54184	1.54184	1.54184
θ range	**3.181 to 71.234°.**	3.181 to 71.234°.	3.181 to 71.234°.	5.515 to 71.224°.
Min./max indices *h, k, l*	**-19/19,-19/19,-9/9**	-19/19,-19/19,-9/9	-19/19,-19/19,-9/9	-11/11,-11/11,-9/9
F(000)	**1080**	1080	1080	360
Reflections all	**37462**	37462	37462	13171
Independent reflections/ R_{int}	**2269/0.0573**	2269/0.0573	1242/0.0584	416/0.0695
θ to 100% Completeness	**67.684°**	67.684°	67.684°	67.684°
Max./min. transmission	**0.334/0.060**	0.334/0.060	0.334/0.060	1.0/0.19097
Data / restraints / parameters	**2269 / 13 / 131**	2269 / 5 / 119	1242 / 21 / 71	416 / 3 / 26
GooF on F^2	**1.053**	1.054	1.181	1.100
Final R_1/wR_2 Σ[I>2σ$_I$]	**0.0287/0.0782**	0.0329/0.0918	0.0648/0.2075	0.0237/0.0600
R_1/ wR_2 (all data)	**0.0319/0.0814**	0.0362/0.0957	0.0703/0.2163	0.0237/0.0600
Extinction coefficient	**0.00143(13)**	0.00120(13)	0.0051(10)	0.00143(13)
Absorption coefficient [mm^{-1}]	**12.368**	12.368	12.368	12.365
Absolute structure parameter	**-0.02(2)**	-0.02(2)	-0.02(2)	-0.02(2)
Max. diff. peak / hole [e. Å^{-3}]	**0.443 / -0.468**	0.932 / -0.519	1.108 / -2.211	0.618 / -0.505

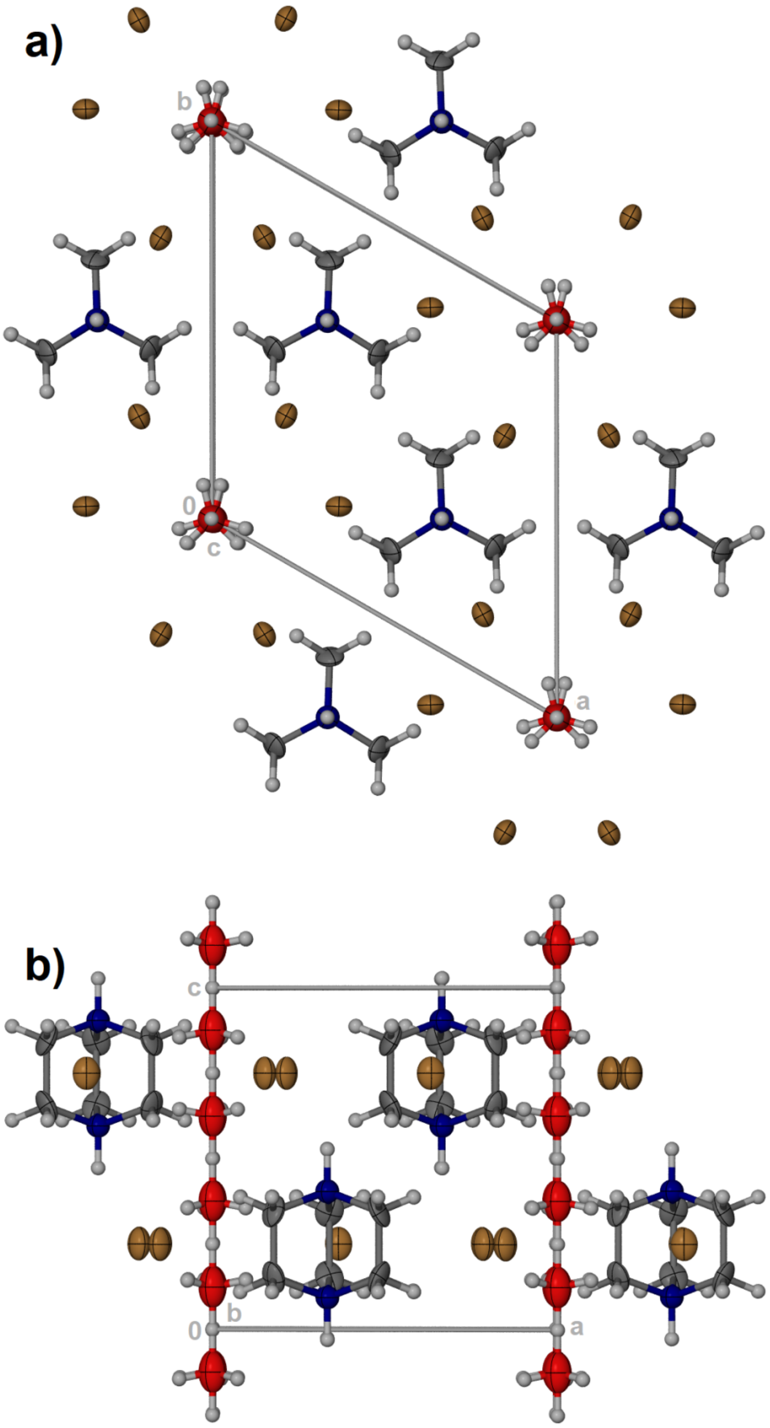

Figure S11 Averaged structure of the pseudo-hexagonal of polymorph β-$[dabcoH_2]^{2+}[H_3O]^{+}Br_3^{-}$ (Model 4 in Table S1) projected along: (a) direction [001]; and (b) along direction [010]. In this pseudo-hexagonal model all hydronium cations $[H_3O]^{+}$ are disordered in the ½-occupied sites by O-atoms and ³/₈-occupied sites of H-atoms (*i.e.* ¼ by H-atoms and ¹/₈ by protons). The colors represent different types of atoms: brown Br, blue N, red O, grey C and light grey H.

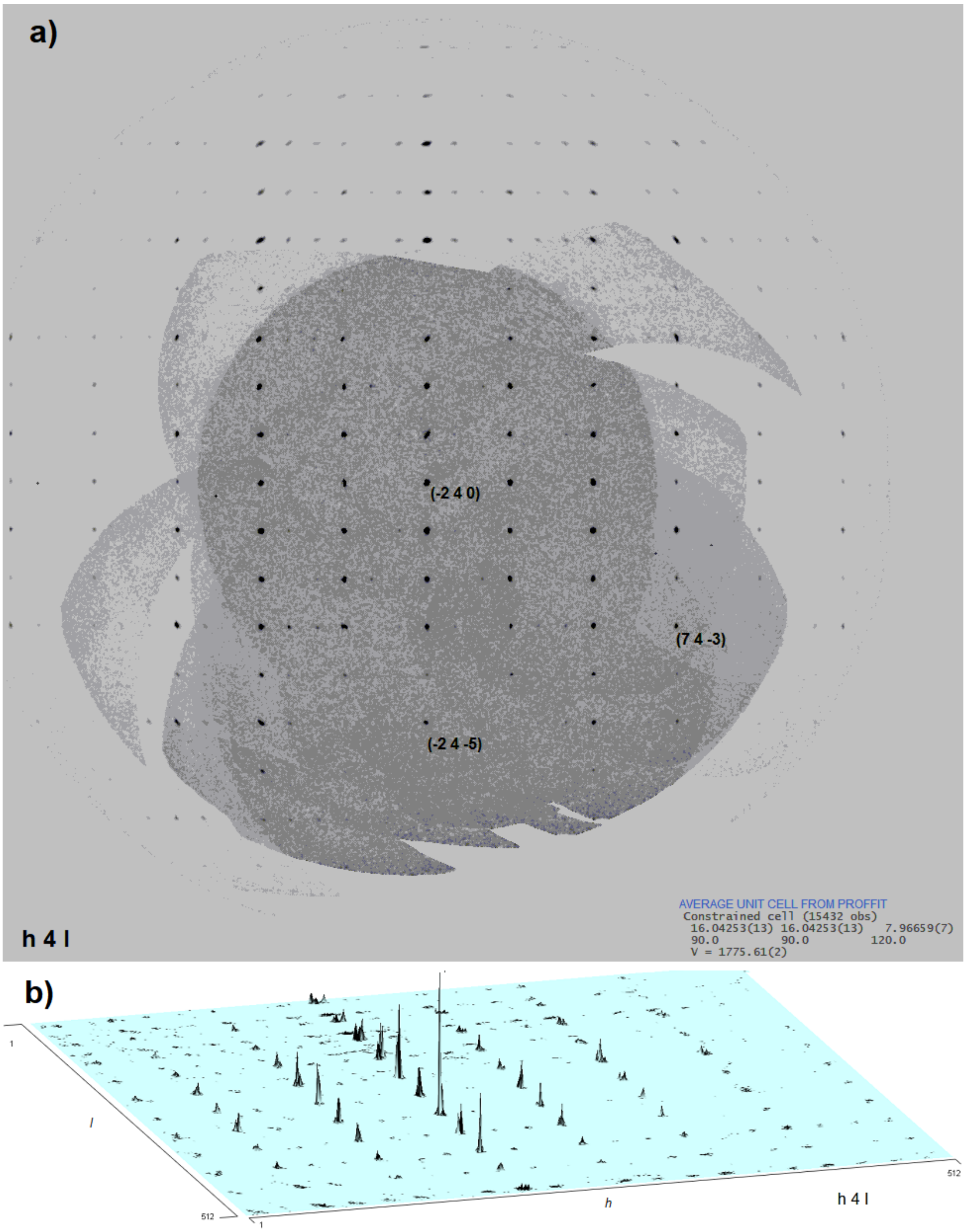

Figure S12 (a) Indexed layer *h*4*l* measurement for polymorph β (Table S1); (b) the 3-D profile (Rigaku Oxford Diffraction, 2021) visualization of intensities of this layer.

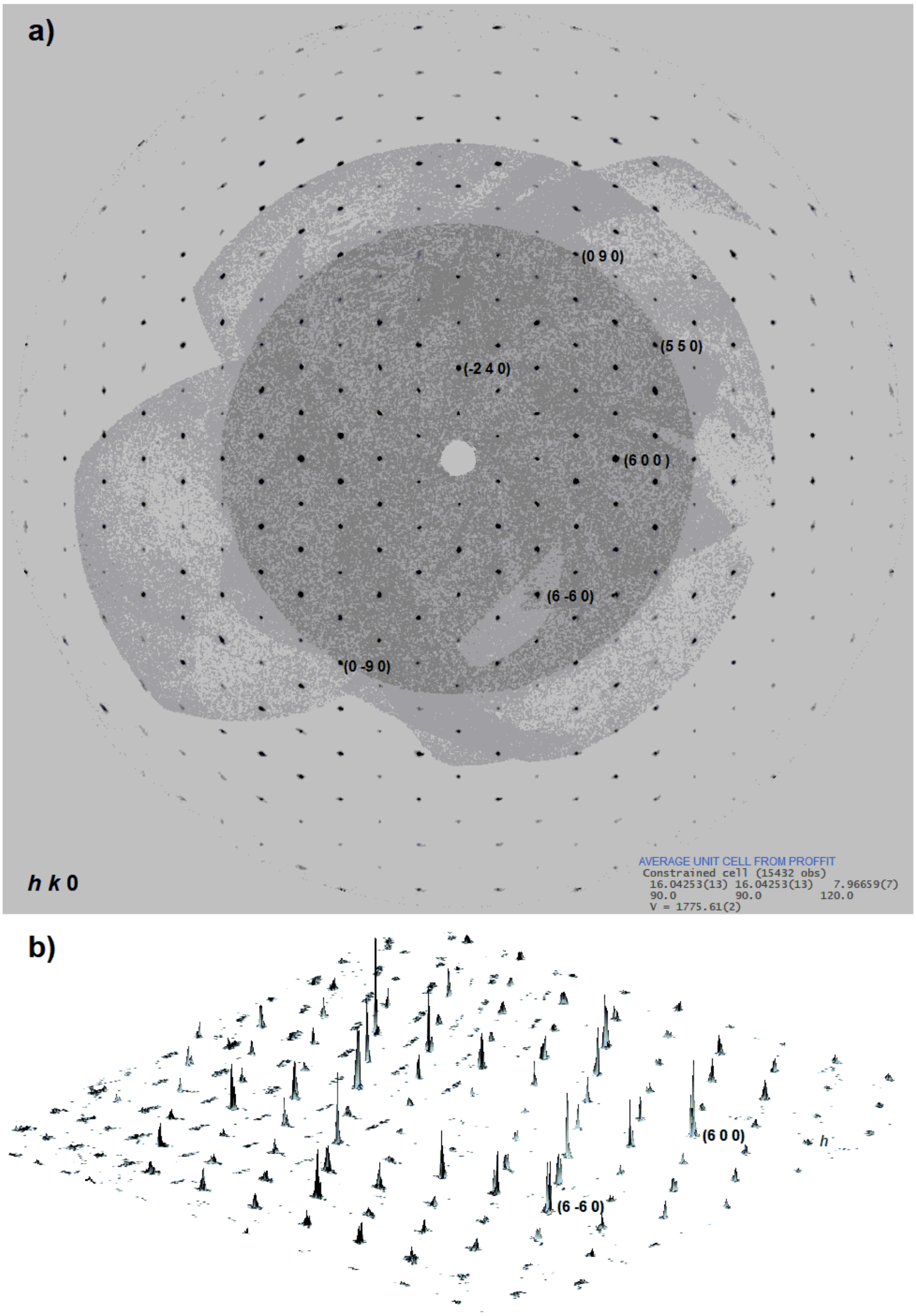

Figure S13 (a) Indexed layer *hk*0 generated measured for polymorph β (Table S1); b) the 3-D profile (Rigaku Oxford Diffraction, 2021) visualization of intensities of the layer.

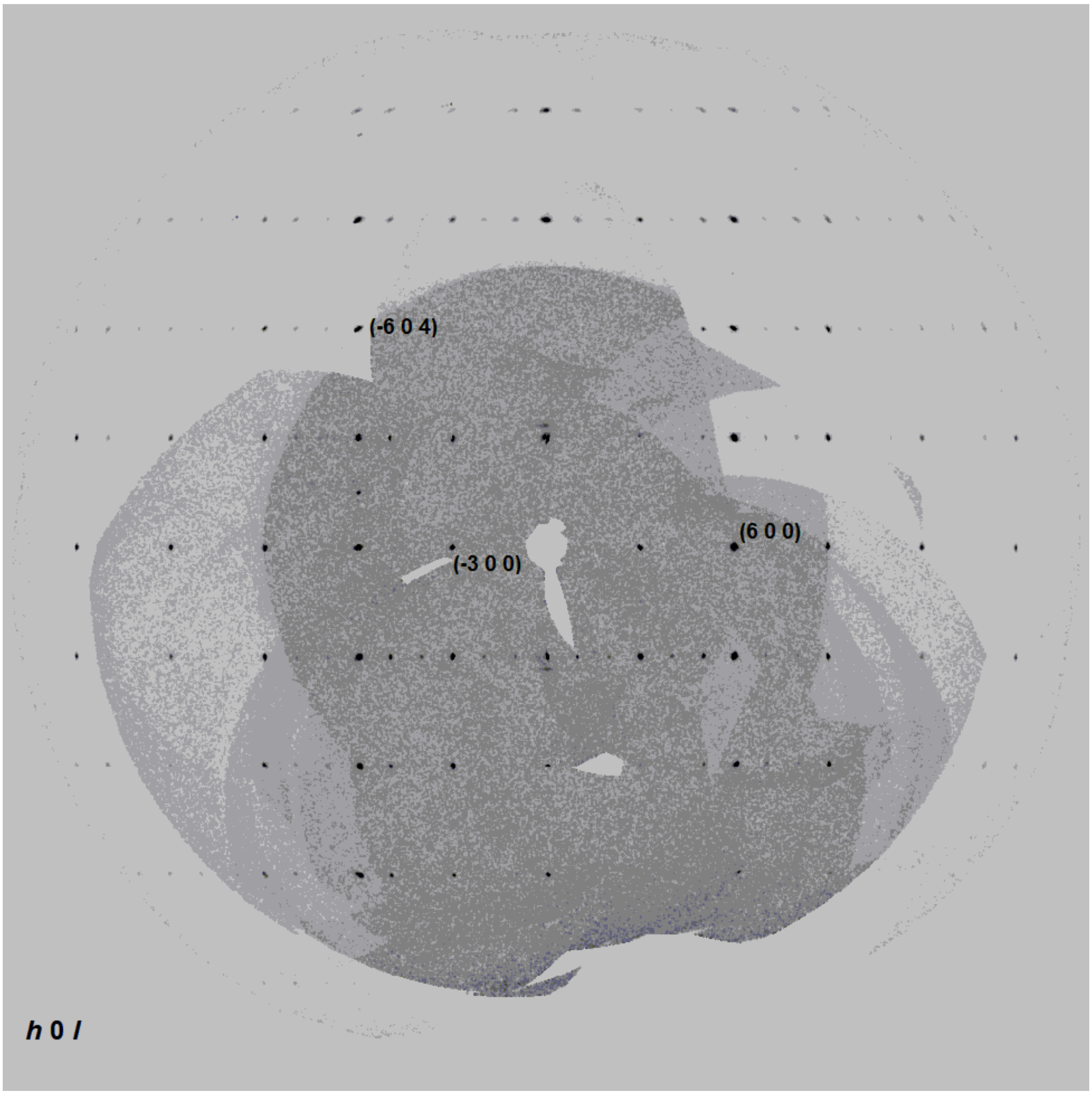

Figure S14 Layer *h*0*l* measured (Rigaku Oxford Diffraction, 2021) for polymorph β (Table S1).

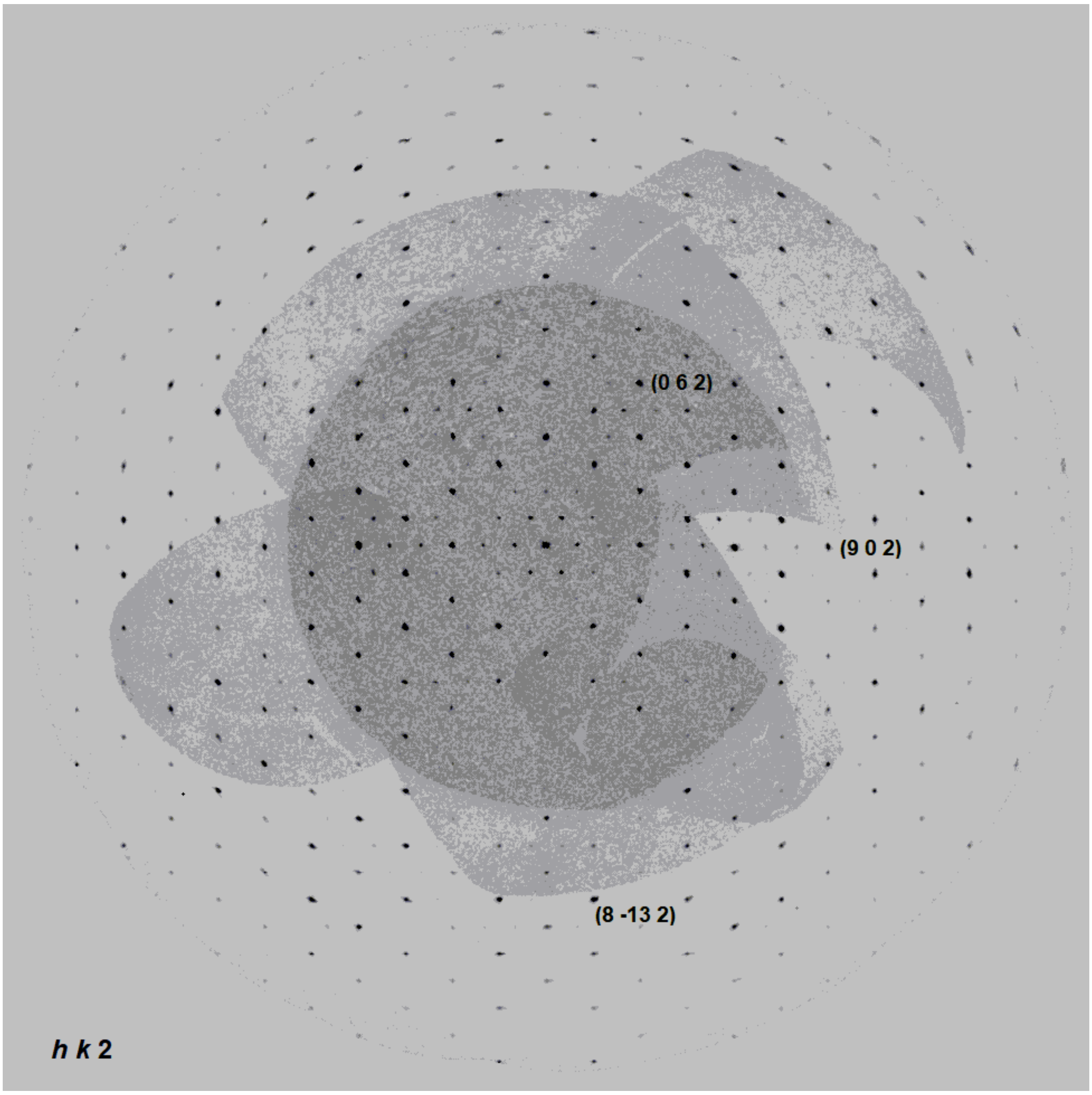

Figure S15 Indexed layer *hk*2 measured (Rigaku Oxford Diffraction, 2021) for polymorph β (Table S1).

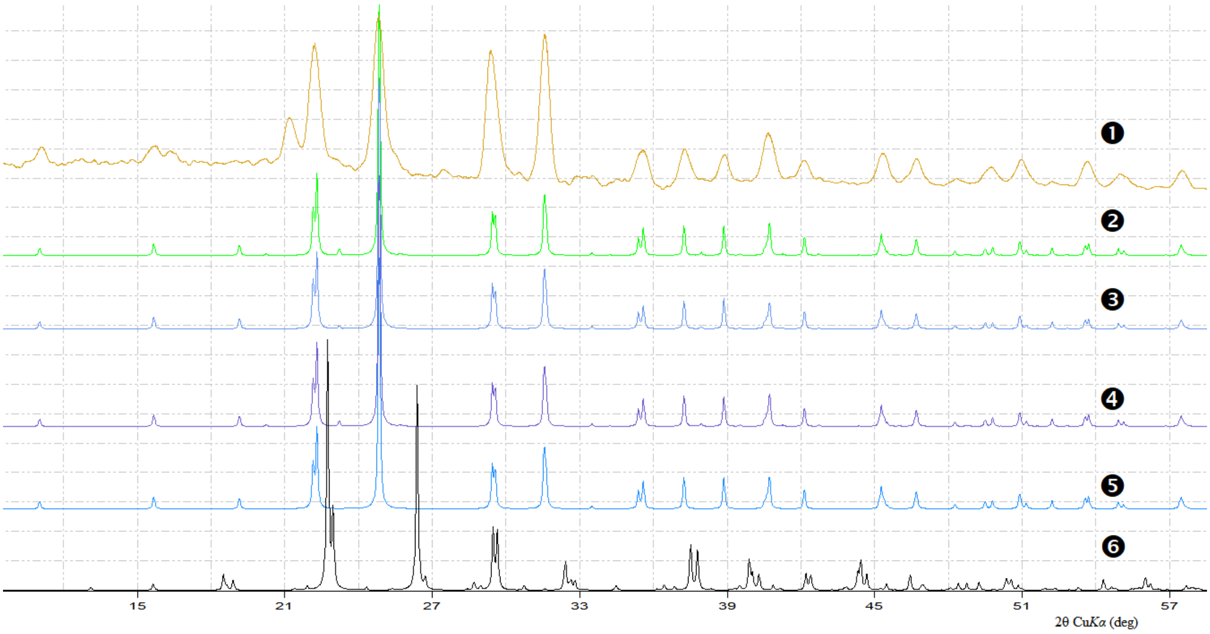

Figure S16 Comparison (Laetsch & Downs, 2006) of powder X-ray diagrams for β-[dabcoH$_2$]$^{2+}$[H$_3$O]$^+$Br$_3^-$ models (patterns 1-5) and for α-[dabcoH$_2$]$^{2+}$[H$_3$O]$^+$Br$_3^-$ (pattern 6). Pattern (1) was extracted (Rigaku Oxford Diffraction, 2019b) from measured single-crystal X-ray diffraction data for polymorph β; next patterns (2-5) were calculated from the structural models of polymorph β listed in Table S1 (β polymorph, Models 2, 3 and 4, respectively).

Table S2 Angles H-O-H (°) of 50:50 disordered hydronium ions in the α-[dabcoH$_2$]$^{2+}$[H$_3$O]$^+$Br$_3^-$ structure. Symmetry codes: (i) *y*, *x*, 1-*z*; (ii) 2-*x*, 1-*x*+*y*, 2/3-*z*.

H-O-H (°)	Site 1 H$_3$O(1w)$^+$		H-O-H (°)	Site 1 H$_3$O(2w)$^+$
108(2)	H(11w)-O(1w)-H(12w)		105(4)	H(21w)-O(2w)-H(22w)
108(2)	H(11w)-O(1w)-H(13w)		107(4)	H(21w)-O(2w)-H(23w)
108(2)	H(12w)-O(1w)-H(13w)		104(4)	H(22w)-O(2w)-H(23w)
	Site 2 H$_3$O(1w)$^+$			Site 2 H$_3$O(2w)$^+$
108(2)	H(11w)^{i}-O(1w)-H(12w)^{i}		105(4)	H(21w)^{ii}-O(2w)-H(22w)^{ii}
108(2)	H(11w)^{i}-O(1w)-H(13w)^{i}		107(4)	H(21w)^{ii}-O(2w)-H(23w)^{ii}
108(2)	H(12w)^{i}-O(1w)-H(13w)^{i}		104(4)	H(22w)^{ii}-O(2w)-H(23w)^{ii}
	Between sites 1 and 2			Between sites 1 and 2
58(6)	H(11w)-O(1w)-H(12w)^{i}		175(8)	H(21w)-O(2w)-H(22w)^{ii}
58(6)	H(12w)-O(1w)-H(11w)^{i}		175(8)	H(22w)-O(2w)-H(21w)^{ii}
165(7)	H(11w)-O(1w)-H(13w)^{i}		81(9)	H(21w)-O(2w)-H(23w)^{ii}
165(7)	H(13w)-O(1w)-H(11w)^{i}		81(9)	H(23w)-O(2w)-H(21w)^{ii}
85(5)	H(12w)-O(1w)-H(13w)^{i}		68(8)	H(22w)-O(2w)-H(23w)^{ii}
85(5)	H(13w)-O(1w)-H(12w)^{i}		68(8)	H(23w)-O(2w)-H(22w)^{ii}
74(8)	H(11w)-O(1w)-H(11w)i		72(10)	H(21w)-O(2w)-H(21w)^{ii}
164(6)	H(12w)-O(1w)-H(12w)^{i}		78(7)	H(22w)-O(2w)-H(22w)^{ii}
75(9)	H(13w)-O(1w)-H(13w)^{i}		170(11)	H(23w)-O(2w)-H(23w)^{ii}

Structural models were drawn in VESTA or in CCDC Mercury or in X-Seed or in POV-Ray software (Momma K. & Izumi, F. 2011; Macrae *et al.*, 2020; Barbour, 2001; Persistence of Vision Pty. Ltd., 2004), some of them as autostereograms (Katrusiak, 2011).

Supplementary References

Bremner, C. A., Simpson, M. & Harrison, W. T. A. (2002). *J. Am. Chem. Soc.* **124**, 10960−10961.

Clark, R. C. & Reid, J. S. (1995). *Acta Cryst. A.* **51**, 887–897.

Katrusiak, A. (2011). Journal of Molecular Graphics and Modelling **19:3–4**, 363–367**.**

Laetsch, T. A. & Downs, R. T. (2006). Program and Abstracts of the 19th General Meeting of the International Mineralogical Association in Kobe, Japan (2006). **P08−25.**

Macrae, C. F., Sovago, I., Cottrell, S. J., Galek, P. T. A., McCabe, P., Pidcock, E., Platings, M., Shields, G. P., Stevens, J. S., Towler M. & Wood, P. A. (2020). *J. Appl. Cryst***. 53,** 226–235.

Momma K. & Izumi, F.(2011). *J. Appl. Crystallogr.* 44, 1272–1276**.**

Persistence of Vision Pty. Ltd. (2004). *Persistence of Vision (TM) Raytracer.* Persistence of Vision Pty. Ltd., Williamstown, Victoria, Australia. http://www.povray.org/.

Rigaku Oxford Diffraction, (2019a). CrysAlisPro 1.171.39.25a.

Rigaku Oxford Diffraction, (2019b). CrysAlisPro 1.171.40.57a.

Rigaku Oxford Diffraction, (2020). CrysAlisPro 1.171.40.79a.

Rigaku Oxford Diffraction, (2021). CrysAlisPro 1.171.41.122a.

Sheldrick, G. M. *(2015). Acta Cryst. C* 71, 3–8.
